# Acute pain in the prehospital setting: a register-based study of 41.241 patients

**DOI:** 10.1186/s13049-018-0521-2

**Published:** 2018-07-03

**Authors:** Kristian D. Friesgaard, Ingunn S. Riddervold, Hans Kirkegaard, Erika F. Christensen, Lone Nikolajsen

**Affiliations:** 1 Research Department, Prehospital Emergency Medical Service, Central Denmark Region, Aarhus, Denmark; 2 Department of Anesthesiology, Regional Hospital of Horsens, Horsens, Denmark; 3 Department of Clinical Medicine, Center for Prehospital and Emergency Research, Aalborg University, Aalborg, Denmark; 4 Department of Anesthesiology and Intensive Care, Emergency Clinic Aalborg University Hospital, Aalborg, Denmark; 5 Prehospital Emergency Medical Services, North Denmark Region, Aalborg, Denmark; 6 Department of Anesthesiology, Aarhus University Hospital, Aarhus, Denmark

**Keywords:** Acute pain, Prehospital, Causes of acute pain

## Abstract

**Background:**

Acute pain is a frequent symptom, but little is known about the frequency and causes of acute pain in the prehospital population. The objectives of this study were to investigate the frequency of moderate to severe pain among prehospital patients and the underlying causes according to primary hospital diagnose codes.

**Methods:**

This was a register-based study on 41.241 patients transported by ambulance. Information on moderate to severe pain [Numeric Rating Scale (NRS, 0–10) > 3 or moderate pain or higher on 4-point likert scale] was extracted from a national electronic prehospital patient record. Patient information was merged with primary hospital diagnose codes based on the 10th version of the International Classification of Diseases (ICD-10) to investigate underlying causes of pain.

**Results:**

11.430 patients (27.7%) reported moderate to severe pain during ambulance transport. As a measure of opioid demanding acute pain, 3.275 of 41.241 patients (7.9%) were treated with intravenous fentanyl. Underlying causes of pain were heterogenic according to ICD-10 chapters with injuries being the largest group of patients with moderate to severe pain (XIX: 42.8% of 8.041 patients), followed by non-specific diagnoses (XVIII: 28.5% of 7.101 patients and XXI: 31.6% of 5.148 patients), diseases of the circulatory system (IX: 22.1% of 4.812 patients) and other (20.3% of 16.139 miscellaneous patients).

**Discussion:**

Due to the high frequency of moderate to severe pain affecting a wide range of patients, more attention on acute pain is necessary. Whether ambulance personnel have sufficient options for treating various pain conditions might be a subject of future evaluation. Non-specific diagnoses accounted for surprisingly many patients with moderate to severe pain, of which many were treated with intravenous fentanyl. This may be substance of further investigation.

**Conclusions:**

Moderate to severe pain is a highly frequent and probably underestimated symptom among patients transported by ambulance. Underlying causes of pain are heterogenic as described by primary hospital diagnose codes. More focus on the treatment of acute pain is needed.

## Background

Prehospital emergency medical service is an evolving field essential in the initial phase of acute patient care. Focus has traditionally been on highly acute conditions such as cardiac arrest [1, 2], myocardial infarction [3], stroke [4, 5], and major trauma [6]. However, these conditions only represent a minor fraction of prehospital patients; thus focus on more prevalent conditions or symptoms is needed in order to optimize diagnostics and treatments beneficial for larger groups of prehospital patients.

Acute pain is perhaps one of the most frequent symptoms felt by patients in emergency medicine [7–9]. Proper treatment of acute pain in the prehospital phase of acute care is recommended to reduce morbidity, ensure patient wellbeing, and ease transportation from scene to hospital admission [10, 11]. Despite the recognition that acute pain is a priority in prehospital care [12–18], little is known about the frequency and causes among prehospital patients [7–9]. The few existing prehospital studies describing the causes of acute pain categorizes patients into rough symptom-based groups, while the exact underlying diagnose-based diseases remain virtually undescribed. Identifying a more precise cause of acute pain requires a unique individual-level data linkage of prehospital record journals with valid inhospital registries on hospital diagnosis codes. This is possible in Denmark, because all residents are allocated a personal civil registration (CPR) number at birth or immigration [19]. Appreciating that the first step in optimizing prehospital pain management is to acquire basic knowledge of acute pain, we aimed to investigate the frequency of moderate to severe prehospital pain and the causes defined by primary hospital diagnosis codes.

## Methods

### Study design and setting

This is a register-based observational study of acute patients transported by ambulance to hospital in the Central Denmark Region over a 9-month period from 1 August 2015 to 30 April 2016.

The Danish healthcare system is tax-supported with patients having unrestricted access to acute help including prehospital care and ambulance transport. The Central Denmark Region (one of five Danish regions) is a mixed rural/urban area of 13,000 km^2^ and provides healthcare for 1,303,000 individuals representing roughly 25% of the entire Danish population. Acute transports are dispatched through a common Emergency Medical Coordination Centre (EMCC) that receives 1-1-2 emergency calls and immediate requests from general practitioners [20]. The prehospital emergency medical services setup is two-tiered: Emergency medical technicians (EMTs) conduct ground ambulance transports and physicians/anesthesiologists working on a ground mobile emergency care unit are dispatched to potentially life-threatening conditions in a rendezvous model. In certain acute and time-critical conditions, a physician-manned helicopter can be dispatched as well.

In the initial on scene assessment of patients, it is possible to categorize acute pain intensity on a 4-point categorical scale (VRS-4) or an 11-point numeric rating scale (NRS, 0–10). Besides non-pharmacological interventions, such as splinting fractures, prehospital patients with painful conditions can be treated with intravenous fentanyl by EMTs, who are also certified to administer nitroglycerine and acetylsalicylic acid to patients with cardiac chest pain. Prehospital emergency physicians have a wider range of analgesic options including alfentanil and ketamine, but these are primarily used for intubation or in cases of haemodynamically instable patients, who only account for a fraction of the total prehospital population. Repeated assessment of pain intensity on the NRS scale is only required with patients treated with intravenous fentanyl by EMTs.

The most acute patients are transferred to acute hospitals in the region for further diagnostic testing and treatment, or alternatively treated and released on scene after thorough examination by a prehospital emergency physician. These patients may be unharmed victims of traffic accidents, people with treated hypoglycemia, chronic obstructive pulmonary disease or other non-specific complaints assessed as not requiring hospital admission or declared dead on scene arrival [21].

### Study population and outcome

All acute patients transported by ambulance were included. Patients with an unregistered CPR number were excluded. In addition, we did not assess interhospital transports or elective transports with no treatment or monitoring applied. Finally, if the same patient had more than two transports, only the first transport was included.

We primarily assessed the existence of acute moderate to severe pain defined as i) an initial NRS equal to or higher than 4; or ii) ‘moderate’ pain or higher registered on the VRS-4 upon initial patient triage. As a secondary outcome, we explored the presence of severe pain defined as i) initial NRS equal to or higher than 7; or ii) ‘severe’ pain on the VRS-4 [22, 23]. We also explored the frequency of administered intravenous fentanyl as a proxy of opioid demanding pain.

Information on dispatch was extracted from technical dispatch software at the EMCC level [20, 24] and information on patients transported by ambulance was obtained from an electronic touchscreen-based medical record (EMR). We attained additional follow-up data for acute patients transferred to the hospital. The Danish National Patient Registry contains primary and secondary diagnoses according to the 10th version of the International Classification of Diseases (ICD-10), procedures and treatment codes on emergency department and hospital visits since 1995 [25]. The underlying cause of pain was defined by primary hospital diagnosis codes, and presented according to the overall 21 ICD-10 chapters [26]. Primary hospital diagnosis codes were considered reasonable for linkage with EMR data if patients were admitted within 12 h of prehospital (EMCC) contact. To describe the cohort in detail we calculated a 10-year Charlson Comorbidity Index (CCI) score from the extracted ICD-codes [27] and vital status was obtained from the Danish Civil Registration System [19].The Danish Data Protection Agency (no. 1-16-02-294-16) and the National Board of Health (no. 3-3013-1663/1) approved the study. According to Danish law, observational studies do not require informed patient consent and/or approval from local ethics committees.

### Data analysis and handling

All statistical analyses were conducted using STATA version 13.1 (StataCorp, TX, USA). Categorical data was reported as number (%) with 95% confidence intervals (CI), and means with CI were given for continuous data following a normal distribution. A χ^2^ test, log rank test or one-way analysis of variance was used for descriptive purposes when appropriate.

## Results

**Fig. 1:**
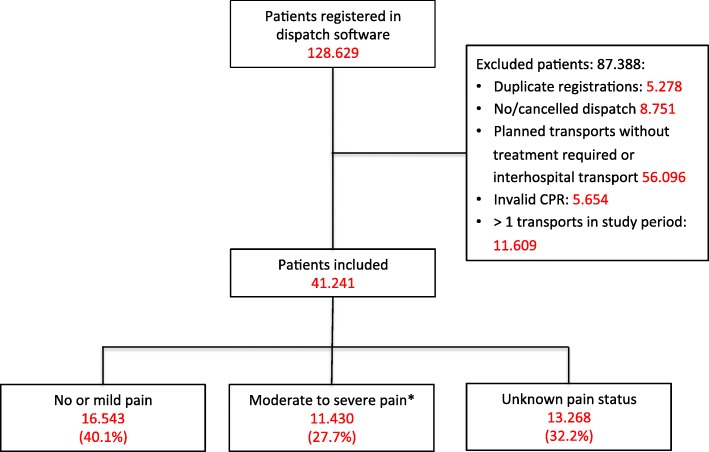
Flow diagram of included patients. Definition of pain on a numeric rating scale (NRS, 0–10) or 4-point verbal rating scale (VRS-4) upon initial patient triage. No or mild pain: NRS < 4 or less than moderate on VRS-4. Moderate to severe pain: NRS ≥ 4 or at least moderate on VRS-4

**Fig. 2:**
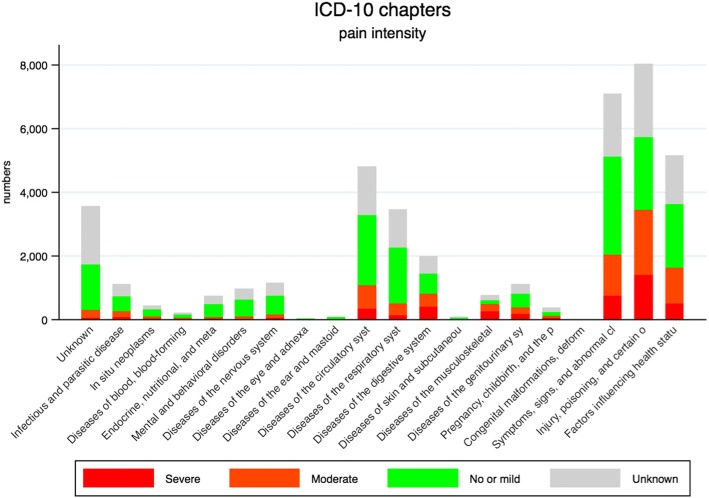
Distribution of pain within the 21 ICD-10 chapters. Definition of pain on a numeric rating scale (NRS, 0–10) or 4-point verbal rating scale (VRS-4) upon initial patient triage. No or mild pain: NRS < 4 or less than moderate on VRS-4. Moderate pain: NRS 4–6 or moderate on VRS-4. Severe pain: NRS ≥ 7 or severe on VRS-4. Abbreviation: ICD-10; 10th version of the International Classification of Diseases

Figure 1 presents the flow of patients. Patient age was 58.6 years (95% CI 58.4–58.8) and 51.1% (95% CI 50.6–51.6) were male. Of the 41.241 acute patients transported to hospital by ambulance, 11.430 [27.7% (95% CI 27.3–28.1)] had moderate to severe pain, 16.543 [40.1% (95% CI 39.6–40.6)] had no or mild pain, and 13.268 [32.2% (95% CI 31.7–32.6)] had no information on pain status. Patients with severe pain (*n* = 4.234) accounted for 10.3% (95% CI 10.0–10.6) of the entire population. The distribution of pain can be seen in Fig. 2 and the proportion of patients receiving fentanyl within the 21 ICD-10 chapters is presented in Table 1. Patients differed substantially between the overall ICD-10 chapters as regards to total number and proportion of cases with moderate to severe pain:

**Table 1:** Distribution of moderate to severe pain and frequency of fentanyl administration within the 21 ICD-10 chapters

	ICD-10 chapters	Total	Moderate to severe pain^a, b^		Fentanyl adm.^b^	
		*n*	Yes (%)	No information (%)	*n*	Yes (%)
I	Infectious and parasitic diseases	1108	22.0 (19.6–24.6)	34.0 (31.2–36.9)	32	2.9 (2.0–4.1)
II	In situ neoplasms	444	21.6 (17.9–25.7)	31.1 (26.8–35.6)	17	3.8 (2.2–6.1)
III	Diseases of blood, blood-forming organs and certain disorders involving the immune mechanism	219	18.3 (13.4–24.0)	29.7 (23.7–36.2)	13	5.9 (3.2–9.9)
IV	Endocrine, nutritional, and metabolic disease	741	10.4 (8.3–12.8)	34.4 (31.0–38.0)	8	1.1 (0.5–2.1)
V	Mental and behavioral disorders	968	8.3 (6.6–10.2)	34.9 (31.9–38.0)	5	0.5 (0.2–1.2)
VI	Diseases of the nervous system	1153	13.6 (11.7–15.7)	35.0 (32.3–37.9)	16	1.4 (0.8–2.2)
VII	Diseases of the eye and adnexa	24	20.8 (7.1–42.2)	25.0 (9.8–46.7)	0	–
VIII	Diseases of the ear and mastoid process	98	14.3 (8.0–22.8)	29.6 (20.8–39.7)	2	2.5 (0.2–7.2)
IX	Diseases of the circulatory system	4812	22.1 (20.9–23.3)	32.0 (30.7–33.4)	356	7.4 (6.7–8.2)
X	Diseases of the respiratory system	3467	14.2 (13.0–15.4)	35.2 (33.7–36.9)	88	2.5 (2.0–3.1)
XI	Diseases of the digestive system	1999	40.7 (38.6–42.9)	28.0 (26.1–30.0)	224	11.2 (9.9–12.7)
XII	Diseases of skin and subcutaneous tissue	96	12.5 (6.6–20.8)	39.6 (29.7–50.1)	0	–
XIII	Diseases of the musculoskeletal system and connective tissue	772	60.6 (57.0–64.1)	23.1 (20.1–26.2)	191	24.7 (21.7–27.9)
XIV	Diseases of the genitourinary system	1105	33.5 (30.7–36.4)	27.4 (24.8–30.2)	124	11.2 (9.4–13.2)
XV	Pregnancy, childbirth, and the puerperium	377	30.2 (25.6–35.1)	39.3 (34.3–44.4)	12	3.2 (1.7–5.5)
XVI	Certain conditions originating in the perinatal period	0	–	–	0	–
XVII	Congenital malformations, deformations, and chromosomal abnormalities	10	–	–	0	–
XVIII	Symptoms, signs, and abnormal clinical and laboratory findings, not elsewhere classified	7101	28.5 (27.4–29.5)	28.1 (27.0–29.1)	355	5.0 (4.5–5.5)
XIX	Injury, poisoning, and certain other consequences of external causes	8041	42.8 (41.7–43.9)	28.8 (27.8–29.8)	1456	18.1 (17.3–19.0)
XX	External causes of morbidity and mortality	0	–	–		–
XXI	Factors influencing health status and contact with health services	5148	31.6 (30.3–32.9)	29.6 (28.4–30.9)	364	7.1 (6.4–7.8)
–	Unknown	3558	8.1 (7.2–9.0)	51.3 (49.7–53.0)	12	0.3 (0.2–0.6)
	Total	41,241	27.7 (27.3–28.2)	32.2 (31.7–32.6)	3275	7.9 (7.7–8.2)

*Abbreviations*: *ICD-10* 10th version of the international classification of diseases, *Adm* administration

^a^Moderate to severe pain defined as a numeric rating scale ≥ 4 or moderate pain or higher registered on a 4-point verbal rating scale upon initial patient triage

^b^Given as proportions (**%**) with 95% confidence intervals

The ICD-10 chapters with the highest *number* of patients with moderate to severe pain were chapter XIX ‘Injury, poisoning, and certain other consequences of external causes’ (*n* = 3.442) followed by non-specific XVIII ‘Symptoms, signs, and abnormal clinical and laboratory findings, not elsewhere classified’ (*n* = 2.022) and XXI ‘Factors influencing health status and contact with health services’ (*n* = 1.630), and lastly IX ‘Diseases of the circulatory System’ (*n* = 1.064) (Table 2).

**Table 2:** Frequency of fentanyl administration among patients with moderate and severe pain

	ICD-10 chapters	Total	Moderate^a, b^	Fentanyl adm.	Severe^a, c^	Fentanyl adm.
		*n*	%	*n* (%)	%	*n* (%)
I	Infectious and parasitic diseases	244	73.0 (66.9–78.4)	8 (3.3)	27.0 (21.6–33.1)	21 (8.6)
II	In situ neoplasms	96	63.0 (52.0–72.2)	3 (3.1)	37.0 (27.8–48.0)	10 (10.4)
III	Diseases of blood, blood-forming organs and certain disorders involving the immune mechanism	40	57.5 (40.9–73.0)	1 (2.5)	42.5 (27.0–59.1)	11 (27.5)
IV	Endocrine, nutritional, and metabolic disease	77	70.1 (58.6–80.0)	2 (2.6)	29.9 (20.0–41.3)	5 (6.5)
V	Mental and behavioral disorders	80	83.8 (73.8–91.1)	–	16.2 (8.9–26.2)	3 (3.8)
VI	Diseases of the nervous system	157	63.7 (55.7–71.2)	3 (1.9)	36.3 (28.8–44.3)	7 (4.5)
VII	Diseases of the eye and adnexa	5	80.0 (28.4–99.5)	–	20.0 (0.5–71.6)	–
VIII	Diseases of the ear and mastoid process	14	64.3 (35.1–87.2)	–	35.7 (12.8–64.9)	2 (14.3)
IX	Diseases of the circulatory system	1064	68.2 (65.3–71.0)	111 (10.4)	31.8 (29.0–34.7)	156 (14.7)
X	Diseases of the respiratory system	491	75.5 (70.4–78.3)	16 (3.2)	24.5 (21.7–29.6)	48 (9.8)
XI	Diseases of the digestive system	814	50.5 (47.0–54.0)	39 (4.8)	49.5 (46.0–53.0)	167 (20.5)
XII	Diseases of skin and subcutaneous tissue	12	83.3 (51.6–97.9)	–	16.7 (2.1–48.4)	–
XIII	Diseases of the musculoskeletal system and connective tissue	468	45.5 (40.9–50.1)	38 (8.1)	54.5 (49.9–59.1)	145 (31.0)
XIV	Diseases of the genitourinary system	370	53.0 (47.7–58.2)	25 (6.8)	47.0 (41.8–52.3)	90 (24.3)
XV	Pregnancy, childbirth, and the puerperium	114	59.6 (50.1–68.7)	3 (2.6)	40.4 (31.3–49.9)	9 (7.9)
XVI	Certain conditions originating in the perinatal period	0	–	–	–	–
XVII	Congenital malformations, deformations, and chromosomal abnormalities	3	–	–	–	–
XVIII	Symptoms, signs, and abnormal clinical and laboratory findings, not elsewhere classified	2022	63.1 (61.0–65.2)	82 (4.1)	36.9 (34.8–39.0)	240 (11.9)
XIX	Injury, poisoning, and certain other consequences of external causes	3442	59.6 (58.0–61.3)	336 (10.0)	40.4 (38.7–42.0)	994 (28.9)
XX	External causes of morbidity and mortality	0	–	–	–	–
XXI	Factors influencing health status and contact with health services	1630	69.8 (67.5–72.0)	118 (7.2)	30.2 (28.0–32.5)	182 (11.2)
–	Unknown	287	85.0 (80.4–88.9)	2 (0.7)	15.0 (11.1–19.6)	8 (2.9)
	Total	11,430	27.7 (27.3–28.2)	788 (6.9)	32.2 (31.7–32.6)	2098 (18.4)

*Abbreviations*: *ICD-10* 10th version of the international classification of diseases, *Adm* administration

^a^Given as proportions (**%**) with 95% confidence intervals

^b^Moderate pain defined as a numeric rating scale 4–6 or moderate pain registered on a 4-point verbal rating scale upon initial patient triage

^c^Severe pain defined as a numeric rating scale ≥ 7 or severe pain registered on a 4-point verbal rating scale upon initial patient triage

When focusing on the highest *proportion* of patients with moderate to severe pain, the ICD-10 chapters were XIII ‘diseases of the musculoskeletal system and connective tissue’ (60.6%), XIX ‘Injury, poisoning, and certain other consequences of external causes’ (42.8%), XI ‘diseases of the digestive system’ (40.7%), and XIV ‘Diseases of the genitourinary system’ (33.5%) (Table 1).

For patients without inhospital information on primary hospital diagnosis codes (*n* = 3.558), 389 patients were declared dead on scene, 623 patients did not want transfer to the hospital, 1.835 patients were treated and left on scene and 711 patients had no specific reason registered in the EMR. Four patients treated and released were given intravenous fentanyl.

As a proxy of severe opioid demanding pain, 3.275 of 41.241 patients [7.9% (95% CI 7.7–8.2)] were treated with intravenous fentanyl, of which 2.886 patients had moderate to severe pain. Patients with the highest levels of pain were more likely to be treated with fentanyl compared with lower levels of pain [severe pain, *n* = 2.098: 49.6% (95% CI 48.0–51.1), moderate pain, *n* = 788: 11.0% (95% CI 10.2–11.7), no or mild pain, *n* = 163: 1.1% (95% CI 0.8–1.1), unknown pain status, *n* = 226: 1.7% (95% CI 1.5–1.9), *P* = 0.001]. Table 2 provides the distribution of moderate and severe pain and the number of patients given fentanyl after omitting patients with no or mild pain and unknown pain status. Generally, ICD-10 chapters with the highest proportion of severe pain had the highest numbers and proportions of patients given fentanyl. As seen in Table 3, patients with different pain status also varied in terms of age, sex, comorbidity, 30-day mortality and type of dispatch. Adding additional transports provided to the same patient in the study period did not change the frequency of moderate to severe pain or the distribution of pain within ICD-10 chapters (Appendix).

**Table 3:** Characteristics of patients according to pain status

	Moderate to severe pain^a, b^			
	Yes	No	No information	
Age^c^	54.4 (54.0–54.8)	61.4 (61.1–61.8)	58.7 (58.2–59.1)	*P* = 0.02
Male sex^b^	46.9 (45.9–47.8)	53.4 (52.7–54.2)	51.9 (51.0–52.7)	*P* = 0.001
Comorbidity^b^				
0	54.8 (53.9–55.7)	40.8 (40.0–41.5)	43.7 (42.9–44.5)	
1	15.9 (15.2–16.6)	20.1 (19.5–20.7)	18.3 (19.5–20.7)	
2	11.1 (10.5–11.7)	13.4 (12.9–13.9)	13.4 (12.8–13.9)	
3+	18.2 (17.4–18.8)	25.7 (25.0–26.4)	24.6 (23.9–25.4)	*P* = 0.001
30 day mortality^b^	2.8 (2.5–3.1)	4.4 (4.1–4.7)	5.8 (5.4–6.2)	*P* = 0.0001
112 emergency call^b^	50.7 (49.7–51.6)	47.0 (46.2–47.8)	52.2 (51.3–53.0)	*P* = 0.001

^a^Moderate to severe pain defined as a numeric rating scale ≥ 4; or “moderate” pain or higher registered on a 4-point verbal rating scale upon initial patient triage

^b^Given as proportions (**%**) with 95% confidence intervals

^c^Given as mean with 95% confidence intervals

## Discussion

In this large prehospital population-based study, moderate to severe pain was experienced by at least 28% of more than 40.000 patients transported by ambulance, 10.3% had severe pain, and 7.9% were treated with intravenous fentanyl. According to primary hospital diagnosis, the underlying cause of pain was heterogenic with pain present in virtually all ICD-10 chapters. As expected, the ICD-10 chapter with the highest *number* of patients with moderate to severe pain was injuries (XIX). Surprisingly, a high number of patients with non-specific (XVIII and XXI) diagnoses had moderate to severe pain. The ICD-10 chapters with the highest *proportion* of moderate to severe pain were diseases of the musculoskeletal system, injuries, digestive- and genitourinary diseases. Few other studies have investigated the frequency and causes of acute prehospital pain and these studies were less accurate in terms of describing missing data and classifying cause of pain.

McLean and colleagues investigated 14.5 million ambulance transports over a 1-year period and found that 20% experienced moderate to severe pain. The most common reasons for hospital admission among patients with pain were injuries (27%), musculoskeletal symptoms (18%), chest pain (18%), digestive symptoms (11%) and respiratory symptoms (7%). Overall, 17% were treated with narcotic analgesics [7], which seems as a high proportion compared with our results and other studies. An Australian ambulance study estimated that 35% of 315.000 patients experienced pain of any intensity during transport, most part were categorized as being of traumatic (40%) or medical (39%) origin. Seven percent of all patients received opioids in the ambulance [8]. A French prehospital study on 2.279 patients encountered by emergency physicians found that 40% experienced pain of any intensity and 8.3% received opioids. The most common tentative diagnoses were cardiac (29%), traumatic (11%), non-specific (9%), gynaecologic/obstetric (4%), or miscellaneous (45%) [9]. All studies had missing data on pain, varying from 15 to 50%, and they did not properly describe the patients with missing data, which creates space for unaddressed selection bias. Depending on study design and patient selection, other prehospital pain studies experienced missing data on pain documentation in the range of 25–80% [28–35]. In our study, 32% of all patients had missing pain data with differences on various characteristics such as age, sex, comorbidity, 30-day mortality and dispatch type. There are probably several explanations for the cases where no pain score is registered: most likely many of these patients did not to suffer from pain, whereas other might have had pain but no documentation of pain scores. Therefore, a cautious approach should be adopted when patient selection is based on whether pain is documented or not.

Acute pain is a complex subjective feeling that may not always be easily quantified by unidimensional tools [36, 37]. Acute pain is an excruciating experience for the inflicted patient and may have a possible impact on the progression of disease, including immobilization, prolonged inhospital length of stay, and a risk of chronic pain development [38]. Therefore the symptom needs to be assessed and handled as soon as possible. Given the high frequency of moderate to severe pain across a wide range of conditions, it is important to evaluate whether prehospital healthcare providers have optimal possibilities for treating acute pain efficiently. In Scandinavia and many other European countries with similar prehospital setups, simple one-drug pain treatment protocols with dosing restriction are applied for ambulance personnel use, whereas an extended analgesic authority is reserved for prehospital emergency physicians [39–45]. This appears rational from a patient safety perspective but with the majority of patients handled solely by non-physician emergency staff, the risk of inadequate pain treatment seems evident.

A high number of patients treated with intravenous fentanyl may reflect the fact that many prehospital patients have opioid demanding pain. There was a surprisingly high number and proportion of patients with moderate to severe pain among patients with non-specific diagnoses (e.g. ICD-10 chapters XVIII and XXI), which could be elucidated in future studies. Also, it may raise concerns whether few of these patients are malingering as part of a drug seeking behavior. While prehospital healthcare providers have to keep on relieving symptoms and not judging patient reliability, the prehospital EMS contribution to drug-seeking behavior may be substance of further investigation. Reports from the United States and Europe suggest an epidemic outbreak of opioid abuse that needs to be taken seriously [46, 47].

In previous prehospital studies on the causes of pain [7–9], the classification of diseases was uncertain or unspecified, thus making direct comparison with our study difficult. To our knowledge, no other prehospital studies have assessed the causes of pain using an ICD-10-based classification of diseases. Other prehospital investigations have assessed the presented symptoms during 1-1-2 calls, and, though classified differently, the most prevalent patient groups are injuries/accidents, non-specific complaints and symptoms of cardiac-, respiratory- and abdominal origin [20, 24]. As in our study, similar overall distributions of ICD-10 chapters on patients transported by ambulance have been found in other prehospital investigations [48–51].

The strength of this register-based study is first of all the sample size merged with validated national registries [19, 25]. Second, the risk of baseline selection is of less concern since all citizens have equal free access to prehospital emergency care.

A number of limitations also need to be addressed. First, missing CPR numbers have hypothetically contributed to selection bias. Given the population of acute prehospital patients with potential acute diseases assessed in a limited timeframe, the risk of being unable to acquire precise information regarding some individuals is practically unavoidable [20, 49, 52–54]. Second, the data validity of the EMR depends on reliable entry and documentation of variables of scientific interest. For our study, one third of patients had no information on pain status, thus reflecting a general problem within the genre of observational pain research but also emphasizing the need for a careful approach when assessing and interpreting data. Our data suggests that at least 28% of patients experience moderate to severe pain in the ambulances but this proportion is likely to be higher. Future research is needed to define variables mandatory for entry in the EMR and to ensure a national consistency in data collection.

Third, using unidimensional pain scales may not precisely reflect the highly nuanced and subjective sensation of pain experienced by acute patients. Baseline pain does not always mirror a treatment demand, and may be influenced by other factors such as anxiety. Though prone to inter-individual variation, simple pain scales are practical clinical tools widely adopted in prehospital- [55], emergency department- [23], and postoperative settings [56]; other more comprehensive quantifications of pain are less feasible in the prehospital environment. The chosen cut-off points for moderate to severe pain are commonly used in a clinical context [22, 23], but can be subject of debate when applied to specific subpopulations. Last, repeated documentation of NRS were mainly used for patients treated with intravenous fentanyl by EMTs. Evaluating changes in pain scores only when treatment is initiated seems reasonable from a clinical point of view, but limits our assessment of the development of pain symptoms during ambulance transport for all prehospital patients with pain.

## Conclusion

In conclusion, moderate to severe pain is a frequent symptom in the prehospital setting, involving at least 28% of all acute patients transported by ambulance and present in nearly all ICD-10 chapters. The highest number of patients with moderate to severe pain occurred within injuries, non-specific diagnoses and diseases of the circulatory system. The highest proportion of moderate to severe pain occurs among patients with diseases of the musculoskeletal system, injuries and diseases of the digestive- and genitourinary system. More attention should be given to the management of acute pain, given the frequency and the broad range of causes in terms of main hospital diagnoses.

## Figures and Tables

**Fig. 3:**
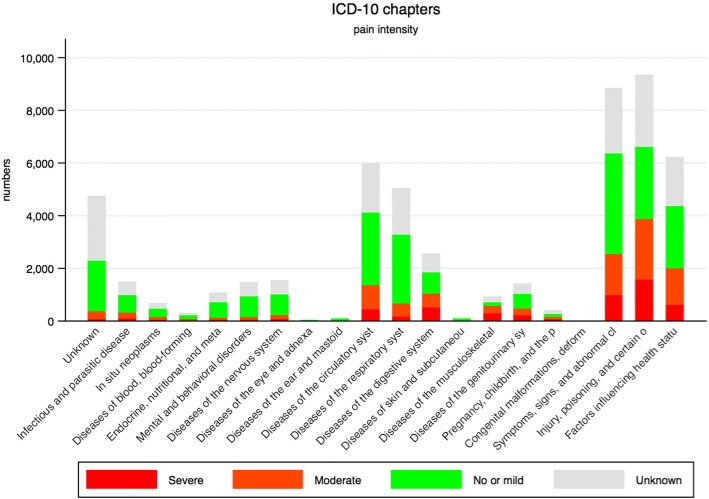
Distribution of pain within the 21 ICD-10 chapters, including additional transports provided to the same patient in the study period. Definition of pain on a numeric rating scale (NRS, 0–10) or 4-point verbal rating scale (VRS-4) upon initial patient triage. No or mild pain: NRS < 4 or less than moderate on VRS-4. Moderate pain: NRS 4–6 or moderate on VRS-4. Severe pain: NRS ≥ 7 or severe on VRS-4. Abbreviation: ICD-10; 10th version of the International Classification of Diseases

## Abbreviations

95 % CI: 95 % Confidence Interval
CCI: Charlson Comorbidity Index
EMCC: Emergency Medical Coordination Centre
EMR: Prehospital electronic medical record
EMT: emergency medical technician
ICD: International Classification of Diseases
NRS: Numeric Rating Scale
VRS-4: 4-point Verbal Rating Scale

## References

[1] Perkins GD, Lall R, Quinn T, Deakin CD, Cooke MW, Horton J (2015). Mechanical versus manual chest compression for out-of-hospital cardiac arrest (PARAMEDIC): a pragmatic, cluster randomised controlled trial. Lancet.

[2] Kudenchuk PJ, Brown SP, Daya M, Rea T, Nichol G, Morrison LJ (2016). Amiodarone, lidocaine, or placebo in out-of-hospital cardiac arrest. N Engl J Med.

[3] Steg PG, van ’t Hof A, Hamm CW, Clemmensen P, Lapostolle F, Coste P (2013). Bivalirudin started during emergency transport for primary PCI. N Engl J Med.

[4] Fassbender K, Grotta JC, Walter S, Grunwald IQ, Ragoschke-Schumm A, Saver JL (2017). Mobile stroke units for prehospital thrombolysis, triage, and beyond: benefits and challenges. Lancet Neurol.

[5] Saver JL, Starkman S, Eckstein M, Stratton SJ, Pratt FD, Hamilton S (2015). Prehospital use of magnesium sulfate as neuroprotection in acute stroke. N Engl J Med.

[6] Pickering A, Cooper K, Harnan S, Sutton A, Mason S, Nicholl J (2015). Impact of prehospital transfer strategies in major trauma and head injury: systematic review, meta-analysis, and recommendations for study design. J Trauma Acute Care Surg.

[7] McLean SA, Maio RF, Domeier RM (2002). The epidemiology of pain in the prehospital setting. Prehosp Emerg Care.

[8] Jennings PA, Cameron P, Bernard S (2011). Epidemiology of prehospital pain: an opportunity for improvement. Emerg Med J.

[9] Galinski M, Ruscev M, Gonzalez G, Kavas J, Ameur L, Biens D (2010). Prevalence and management of acute pain in prehospital emergency medicine. Prehosp Emerg Care.

[10] McManus JG, Sallee DR (2005). Pain management in the prehospital environment. Emerg Med Clin North Am.

[11] Studnek JR, Fernandez AR, Vandeventer S, Davis S, Garvey L (2013). The association between patients’ perception of their overall quality of care and their perception of pain management in the prehospital setting. Prehosp Emerg Care.

[12] Cousins MJ, Lynch ME (2011). The declaration Montreal: access to pain management is a fundamental human right. Pain.

[13] Alonso-Serra HM, Wesley K (2003). Prehospital pain management. Prehosp Emerg Care.

[14] Maio RF, Garrison HG, Spaite DW, Desmond JS, Gregor MA, Cayten CG (1999). Emergency medical services outcomes project I (EMSOP I): prioritizing conditions for outcomes research. Ann Emerg Med.

[15] Spaite DW, Maio R, Garrison HG, Desmond JS, Gregor MA, Stiell IG (2001). Emergency medical services outcomes project (EMSOP) II: developing the foundation and conceptual models for out-of-hospital outcomes research. Ann Emerg Med.

[16] Garrison HG, Maio RF, Spaite DW, Desmond JS, Gregor MA, O’Malley PJ (2002). Emergency medical services outcomes project III (EMSOP III): the role of risk adjustment in out-of-hospital outcomes research. Ann Emerg Med.

[17] Maio RF, Garrison HG, Spaite DW, Desmond JS, Gregor MA, Stiell IG (2002). Emergency medical services outcomes project (EMSOP) IV: pain measurement in out-of-hospital outcomes research. Ann Emerg Med.

[18] Gausche-Hill M, Brown KM, Oliver ZJ, Sasson C, Dayan PS, Eschmann NM (2014). An evidence-based guideline for prehospital analgesia in trauma. Prehosp Emerg Care.

[19] Schmidt M, Pedersen L, Sorensen HT (2014). The Danish civil registration system as a tool in epidemiology. Eur J Epidemiol.

[20] Andersen MS, Johnsen SP, Sorensen JN, Jepsen SB, Hansen JB, Christensen EF (2013). Implementing a nationwide criteria-based emergency medical dispatch system: a register-based follow-up study. Scand J Trauma Resusc Emerg Med.

[21] Hojfeldt SG, Sorensen LP, Mikkelsen S (2014). Emergency patients receiving anaesthesiologist-based pre-hospital treatment and subsequently released at the scene. Acta Anaesthesiol Scand.

[22] Breivik H, Borchgrevink PC, Allen SM, Rosseland LA, Romundstad L, Hals EK (2008). Assessment of pain. Br J Anaesth.

[23] Platts-Mills TF, Esserman DA, Brown DL, Bortsov AV, Sloane PD, McLean SA (2012). Older US emergency department patients are less likely to receive pain medication than younger patients: results from a national survey. Ann Emerg Med.

[24] Moller TP, Ersboll AK, Tolstrup JS, Ostergaard D, Viereck S, Overton J (2015). Why and when citizens call for emergency help: an observational study of 211,193 medical emergency calls. Scand J Trauma Resusc Emerg Med.

[25] Schmidt M, Schmidt SA, Sandegaard JL, Ehrenstein V, Pedersen L, Sorensen HT (2015). The Danish National Patient Registry: a review of content, data quality, and research potential. Clin Epidemiol.

[26] International Statistical Classification of Diseases and Related Health Problems 10th Revision. http://apps.who.int/classifications/icd10/browse/2016/en.

[27] Sundararajan V, Henderson T, Perry C, Muggivan A, Quan H, Ghali WA (2004). New ICD-10 version of the Charlson comorbidity index predicted in-hospital mortality. J Clin Epidemiol.

[28] Albrecht E, Taffe P, Yersin B, Schoettker P, Decosterd I, Hugli O (2013). Undertreatment of acute pain (oligoanalgesia) and medical practice variation in prehospital analgesia of adult trauma patients: a 10 yr retrospective study. Br J Anaesth.

[29] Eidenbenz D, Taffe P, Hugli O, Albrecht E, Pasquier M (2016). A two-year retrospective review of the determinants of pre-hospital analgesia administration by alpine helicopter emergency medical physicians to patients with isolated limb injury. Anaesthesia.

[30] Young MF, Hern HG, Alter HJ, Barger J, Vahidnia F (2013). Racial differences in receiving morphine among prehospital patients with blunt trauma. J Emerg Med.

[31] Platts-Mills TF, Hunold KM, Weaver MA, Dickey RM, Fernandez AR, Fillingim RB (2013). Pain treatment for older adults during prehospital emergency care: variations by patient gender and pain severity. J Pain.

[32] Spilman SK, Lechtenberg GT, Hahn KD, Fuchsen EA, Olson SD, Swegle JR (2016). Is pain really undertreated? Challenges of addressing pain in trauma patients during prehospital transport and trauma resuscitation. Injury.

[33] Middleton PM, Simpson PM, Sinclair G, Dobbins TA, Math B, Bendall JC (2010). Effectiveness of morphine, fentanyl, and methoxyflurane in the prehospital setting. Prehosp Emerg Care.

[34] HHaley KB, Lerner EB, Guse CE, Pirrallo RG. Effect of System-Wide Interventions on the Assessment and Treatment of Pain by Emergency Medical Services Providers. Prehosp Emerg Care. 2016;20(6):752-8. 10.1080/10903127.2016.1182599 27192662

[35] Hewes HA, Dai M, Mann NC, Baca T, Taillac P. Prehospital Pain Management: Disparity By Age and Race. Prehosp Emerg Care. 2018; 22(2):189-97. 10.1080/10903127.2017.1367444 28956669

[36] Jones GE, Machen I (2003). Pre-hospital pain management: the paramedics’ perspective. Accid Emerg Nurs.

[37] Walsh B, Cone DC, Meyer EM, Larkin GL (2013). Paramedic attitudes regarding prehospital analgesia. Prehosp Emerg Care.

[38] Carr DB, Goudas LC (1999). Acute pain. Lancet.

[39] Marinangeli F, Narducci C, Ursini ML, Paladini A, Pasqualucci A, Gatti A (2009). Acute pain and availability of analgesia in the prehospital emergency setting in Italy: a problem to be solved. Pain Pract.

[40] Langhelle A, Lossius HM, Silfvast T, Bjornsson HM, Lippert FK, Ersson A (2004). International EMS systems: the Nordic countries. Resuscitation.

[41] Luiz T, Scherer G, Wickenkamp A, Blaschke F, Hoffmann W, Schiffer M (2015). Prehospital analgesia by paramedics in Rhineland-palatinate: Feasability, analgesic effectiveness and safety of intravenous paracetamol. Anaesthesist.

[42] Roessler M, Zuzan O (2006). EMS systems in Germany. Resuscitation.

[43] Adnet F, Lapostolle F (2004). International EMS systems: France. Resuscitation.

[44] Gomes E, Araujo R, Soares-Oliveira M, Pereira N (2004). International EMS systems: Portugal. Resuscitation.

[45] Papaspyrou E, Setzis D, Grosomanidis V, Manikis D, Boutlis D, Ressos C (2004). International EMS systems: Greece. Resuscitation.

[46] Han B, Compton WM, Blanco C, Crane E, Lee J, Jones CM (2017). Prescription opioid use, misuse, and use disorders in U.S. adults: 2015 National Survey on drug use and health. Ann Intern Med.

[47] Mounteney J, Giraudon I, Denissov G, Griffiths P (2015). Fentanyls: are we missing the signs? Highly potent and on the rise in Europe. Int J Drug Policy.

[48] Christensen EF, Bendtsen MD, Larsen TM, Jensen FB, Lindskou TA, Holdgaard HO (2017). Trends in diagnostic patterns and mortality in emergency ambulance service patients in 2007-2014: a population-based cohort study from the North Denmark region. BMJ Open.

[49] Christensen EF, Larsen TM, Jensen FB, Bendtsen MD, Hansen PA, Johnsen SP (2016). Diagnosis and mortality in prehospital emergency patients transported to hospital: a population-based and registry-based cohort study. BMJ Open.

[50] Kruger AJ, Lossius HM, Mikkelsen S, Kurola J, Castren M, Skogvoll E (2013). Pre-hospital critical care by anaesthesiologist-staffed pre-hospital services in Scandinavia: a prospective population-based study. Acta Anaesthesiol Scand.

[51] Mikkelsen S, Lossius HM, Toft P, Lassen AT (2017). Characteristics and prognoses of patients treated by an anaesthesiologist-manned prehospital emergency care unit. A retrospective cohort study. BMJ Open.

[52] Botker MT, Stengaard C, Andersen MS, Sondergaard HM, Dodt KK, Niemann T (2016). Dyspnea, a high-risk symptom in patients suspected of myocardial infarction in the ambulance? A population-based follow-up study. Scand J Trauma Resusc Emerg Med.

[53] Botker MT, Terkelsen CJ, Sorensen JN, Jepsen SB, Johnsen SP, Christensen EF, et al. Long-Term Mortality of Emergency Medical Services Patients. Ann Emerg Med. 2017; 70(3):366-73. 10.1016/j.annemergmed.2016.12.017 28347554

[54] Moller TP, Kjaerulff TM, Viereck S, Ostergaard D, Folke F, Ersboll AK (2017). The difficult medical emergency call: a register-based study of predictors and outcomes. Scand J Trauma Resusc Emerg Med.

[55] Jennings PA, Cameron P, Bernard S (2009). Measuring acute pain in the prehospital setting. Emerg Med J.

[56] Chou R, Gordon DB, de Leon-Casasola OA, Rosenberg JM, Bickler S, Brennan T (2016). Management of Postoperative Pain: a clinical practice guideline from the American pain society, the American Society of Regional Anesthesia and Pain Medicine, and the American Society of Anesthesiologists’ committee on regional anesthesia, executive committee, and administrative council. J Pain.

